# Results of Magnetic Sphincter Augmentation for Gastroesophageal Reflux Disease

**DOI:** 10.1007/s00268-018-4608-8

**Published:** 2018-04-04

**Authors:** Katrin Schwameis, Milena Nikolic, Deivis G. Morales Castellano, Ariane Steindl, Sarah Macheck, Ivan Kristo, Barbara Zörner, Sebastian F. Schoppmann

**Affiliations:** Department of Surgery, Division of General Surgery, Medical University of Vienna, Waehringer Guertel 18-20, Vienna, 1090 Austria

## Abstract

**Background:**

Magnetic sphincter augmentation (MSA) is a modern treatment option for gastroesophageal reflux disease (GERD); however, laparoscopic fundoplication remains the gold standard. The aim of the study was to evaluate outcomes of MSA patients at a reflux center.

**Methods:**

A retrospective review was performed of all patients that underwent MSA between March 2012 and November 2017. Out of 110 patients, 68 with a follow-up >3 months were included. Postoperative gastrointestinal symptoms, proton pump inhibitor (PPI) intake, GERD-Health-related Quality of Life (GERD-HRQL) and alimentary satisfaction (AS) were assessed. Postoperative esophageal functioning tests were performed in 50% of patients.

**Results:**

Sixty-eight patients underwent MSA; hiatal repair was performed in 31 cases. The median OR time was 27 min, and no intraoperative complications occurred. The median follow-up was 13 months (IQR 4.2–45). Endoscopic dilatation was performed in 2 patients (3%) and device removal in another 2 cases. The postoperative GERD-HRQL score was significantly reduced (3 vs. 24; *p* < 0.001) and the median AS was 8/10. Preoperative experienced heartburn, regurgitations and dysphagia were eliminated in 92, 96 and 100%. Postoperative new-onset difficulties swallowing with solids only were reported to occur occasionally by 16% and rarely by 21% of patients. Satisfaction with heartburn relief was 95%, and the overall outcome was rated excellent/good in 89%. PPI dependency was eliminated in 87%. The median total percentage pH < 4 and number of reflux episodes were significantly reduced. Postoperative pH results were negative or slightly above the norm in 79% and 12%, respectively.

**Conclusion:**

Sphincter augmentation results in significantly reduced reflux symptoms, increased GERD-specific Quality of Life and excellent alimentary satisfaction with low perioperative morbidity. This procedure should be considered an excellent alternative to fundoplication in the treatment of GERD.

## Introduction

The incidence of chronic gastroesophageal reflux disease (GERD) is constantly rising, currently affecting up to 25% of the general population [1, 2]. The first-line therapy for GERD consists of acid suppression with proton pump inhibitors (PPIs), while the surgical gold standard is the laparoscopic fundoplication [1, 3, 4]. Complications of untreated reflux disease include the development of a Barrett’s esophagus and possibly adenocarcinoma of the distal esophagus. Proton pump inhibitors (PPIs) effectively reduce symptoms in approximately 60% of patients by increasing the pH of gastric refluxate. However, they do not address the function of the lower esophageal sphincter (LES) and therefore the reflux itself persists, leaving the potential for development of progressive disease [1, 5–8]. It is believed that bile plays a major role in the development of complications in GERD patients. However, further research is necessary to completely understand the consequences of bile reflux in a pH-altered refluxate [9]. To eliminate the risk of esophageal cancer in GERD patients, antireflux surgery has to be initiated prior to the development of an intestinal metaplasia [10, 11].

On the other hand, possible side effects after fundoplication include gas bloat syndrome, a reduced ability to vomit and belch, and dysphagia. Persistent post-Nissen dysphagia and bloating can result in poor quality of life [12]. Furthermore, alteration of the hiatal anatomy following fundoplication and hiatal repair makes it difficult to re-operate if needed.

In an attempt to provide an alternative treatment option for reflux disease with less side effects, a small device for magnetic sphincter augmentation (MSA) was developed. FDA-approved in 2012, the flexible ring (LINX® Reflux Management System; Torax Medical, Maple Grove, MN), consisting of interlinked magnetic titanium beads, serves to augment the weak LES, suppressing reflux episodes while enabling its physiological functions to proceed uninhibited [9].

Within the last years, several studies have proven magnetic sphincter augmentation to be safe and efficient in the treatment of mild to moderate reflux disease. LINX implantation resulted in a reduced dependence on PPIs and improved GERD-specific Quality of Life while leading to a low rate of side effects, thus making it a preferred alternative in the treatment of GERD [4, 13, 14].

This retrospective study evaluates outcomes and Quality of Life in GERD patients following magnetic sphincter augmentation at a specialized reflux center over a five-year period. Furthermore, esophageal functioning tests were performed to objectively assess reflux control provided by MSA.

## Methods

A retrospective chart review was performed of all patients that underwent laparoscopic magnetic sphincter augmentation (MSA) for reflux disease between March 2012 and September 2017 at our institution. A total of 110 patients were treated by MSA within the study duration. Of these, 68 patients with a follow-up time over 3 months were included into the study for further evaluation.

The initial 37 patients had received exclusive magnetic sphincter augmentation and were followed by a consecutive series of 31 patients that had undergone MSA and crural closure.

We performed a detailed review of a prospectively established database which contains preoperative clinical, radiological and histological data, as well as surgical results and outcome parameters.

In addition to postoperative clinic visits, a standardized telephone interview was conducted by the same physician using a specific script. Postoperative gastrointestinal symptoms, proton pump inhibitor (PPI) intake, GERD-Health-related Quality of Life (GERD-HRQL) and alimentary satisfaction (AS) were assessed. Overall alimentary tract comfort was rated from 0 to 10. A score of 10 indicated complete satisfaction, and a score of 0 indicated an intolerable alimentary function [15]. Patients served as their own control when comparing pre- and postoperative scores of validated questionnaires including the GERD-Health-Related Quality of Life (GERD-HRQL) [16–18]. The frequency and severity of postoperative dysphagia was reported based on the classification of Saeed et al. [19]. Patients with a follow-up over 6 months were asked to schedule postoperative esophageal functioning testing consisting of a high-resolution manometry and impedance-pH-metry.

This study was approved by the Institutional Review Board of the Medical University of Vienna, Austria.

### Preoperative evaluation

Preoperative diagnostics included a standardized interview, the performance of an upper GI endoscopy, a video esophagram and esophageal functioning testing consistent of a high-resolution manometry (HRM) and a 24-hour impedance-pH-metry.

All patients underwent preoperative assessment by high-resolution manometry (Sandhill BioView; Sandhill Zvu; Medtronic ManoScan). Manometric findings were reported in accordance with the Chicago classification v3.0.

Once off proton pump inhibitors for 14 days, patients underwent an ambulatory continuous 24-h esophageal impedance-pH-monitoring with a transnasal catheter (Sandhill ComforTec Z/pH ZAN-BS-01/ZAN-BG-44). The pH probes were positioned on the basis of manometry findings 5 cm above the upper border of the lower esophageal sphincter (LES) as previously described [20]. Patients were instructed to precisely document their food and fluid intakes in a diet diary. Analysis of impedance-pH results included the total number of reflux episodes, the percentage time pH < 4 in total, in upright and supine position and postprandial. An abnormal pH test was based on the number of reflux episodes (normal < 73 episodes/24 h) and the total percentage time pH < 4 (normal < 4.2%). Additionally, a symptom correlation analysis was performed.

### Surgery

All procedures were performed by the same surgeon using standard surgical techniques as described previously [21, 22]. Surgical approach was laparoscopic in all cases, and there were no conversions to open surgery. Briefly, after mobilization of the esophagogastric junction the adequate ring size was measured with the sizing tool and the magnetic ring was wrapped around the lower end of the lower esophageal sphincter.

All procedures were standardized regarding surgeon’s and patient’s positions (anti-Trendelenburg), further trocar sites and used instruments.

After the performance of our initial 37 magnetic sphincter augmentations, a consecutive series of 31 patients followed that received additional hiatal repair. These procedures were accomplished by hiatal dissection and crural closure with 2–5 stitches using non-absorbable sutures. All cases were performed without the use of an esophageal bougie.

After the surgery, patients received an unrestricted diet to avoid the development of dysphagia due to scar tissue surrounding the device. After at least one overnight stay, patients were discharged from the hospital once they were eating solid foods and showing an unsuspicious postoperative barium swallow.

Postoperative follow-up visits were scheduled at 3 weeks, 6, 12 months and then each year after surgery.

### Statistical analysis

Statistical analysis was performed using SPSS® statistics 20.0 (IBM, Armonk, NY). Data were described using median (interquartile range) or mean (range). Statistical analysis appropriate for nonparametric data was used. Categorical variables were assessed using the Fisher exact test and continuous data using the Wilcoxon Rank test as appropriate. Statistical significance was defined as a *p* value < 0.05.

## Results

Sixty-eight patients (46 males, 22 females) underwent magnetic sphincter augmentation (MSA) for chronic gastroesophageal reflux disease at a median age of 45 years (IQR 38–58). A hiatal hernia was present in 52 patients with a median size of 2 cm (IQR 1–3) and a maximum size of 6 cm. Most common symptoms prior to surgery were heartburn (96%), regurgitations (68%) and dysphagia (15%). The latter was mostly due to esophagitis. The median preoperative BMI was 25 (IQR 22–29). Preoperative pH results were abnormal in 53 patients (78%). In the remaining 15 patients (22%), indication for surgery was a shortened, hypotensive lower esophageal sphincter (LES) on high-resolution manometry (HRM) and a positive symptom correlation for heartburn detected by impedance-pH-metry. Three patients (4%) showed an ineffective esophageal motility (IEM) on high-resolution manometry prior to surgery. Demographics and preoperative findings are shown in Table 1.

**Table 1:** Demographic data and results of preoperative diagnostics

	Total *n* = 68 (100%)
Sex (m vs. f)	46 (68) vs. 22 (32)
Median age (IQR)	45 (38–58)
Presence of hiatal hernia	52 (77)
Median HH size in cm (IQR)	2 (1–3)
Median BMI* (IQR)	25 (22–29)
Median total # reflux episodes (normal < 73)	67 (52–86)
Median total percentage time pH < 4 (normal < 4.2%)	4.7 (2.6–10.3)
High-resolution manometry	
Normal motility	65 (96)
IEM^†^	3 (4)
Median LES resting pressure (normal 10–45 mmHg)	20 (11.3–25)
Median IRP^‡^ (normal < 20 mmHg)	8 (6–12)
Median GERD-HRQL total score^§^	24 (16–30)

*BMI body mass index; ^†^*IEM* ineffective esophageal motility; ^‡^*IRP* integrated relaxation pressure of LES; ^§^*GERD-HRQL* total score ranges from 0 to 50

The median OR time was 27 min (range 11–55), and no perioperative complications occurred. The device size most frequently used was 15 [12–16]. A crural closure was accomplished in 31 patients (46%) during the LINX implantation. Sixty-eight percent of patients were discharged from the hospital within 48 hours after surgery.

The median follow-up time was 13 months (IQR2 4.2–45). Surgery did not lead to relevant changes in BMI [median 25 (IQR 22–29) vs. 26 (IQR, 23–29), *p* = 0.861]. Endoscopic dilatation was successfully performed in 2 patients (3%) with persistent dysphagia. Device removal (3%) was carried out in another 2 patients: in one case on the third postoperative day due to esophageal spasm and in the other case after 14 months based on the patient`s wish.

**Fig. 1:**
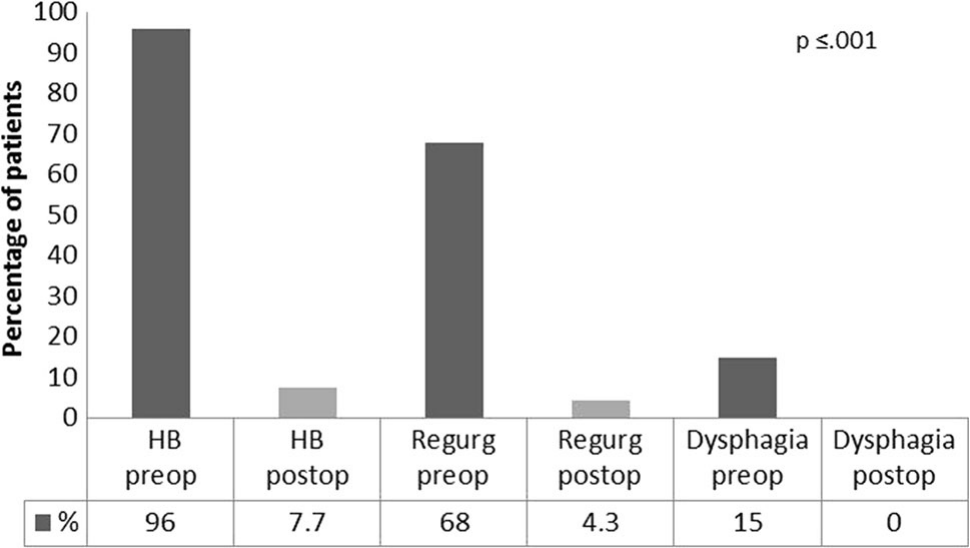
Comparison of pre- and postoperative symptoms (%)

**Fig. 2:**
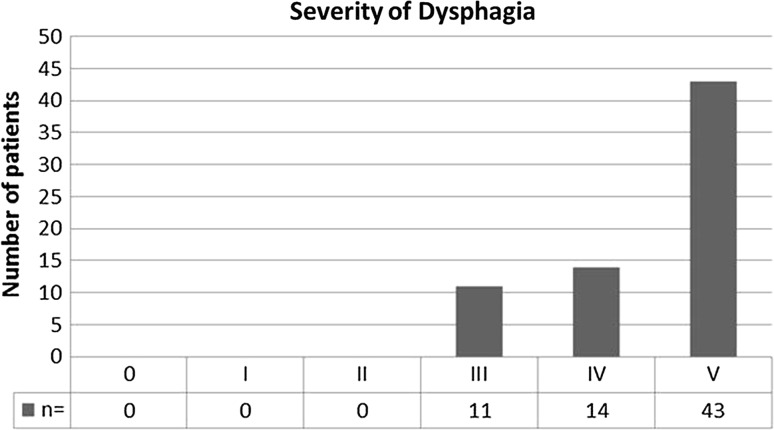
Frequency and degree of postoperative dysphagia based on the classification of Saeed et al. Columns from left to right: 0 = unable to swallow (0%), I = swallowing liquids with difficulty, solids impossible (0%), II =  swallowing liquids without difficulty, solids impossible (0%), III =  occasionally difficulty swallowing with solids (16%), IV = rarely difficulty swallowing with solids (21%), V = swallowing normally (63%)

Preoperative heartburn, regurgitations and dysphagia were eliminated in 92, 96 and 100% of cases, respectively (Table 2). A comparison of pre- and postoperative symptoms is shown in Fig. 1. No patient suffered from persistent dysphagia at the last follow-up. However, postoperative new-onset difficulties swallowing with solids only were reported rarely by 21% (*n* = 14) and occasionally by 16% (*n* = 11) of patients. The frequency and severity of postoperative dysphagia based on the classification of Saeed et al is shown in Fig. 2.

**Table 2:** Postoperative symptom relief

Total *n* = 68 (100%)	Preop. symptoms	Postop. symptoms	Symptom relief	*p* value
Heartburn (HB)	65 (96)	5/65 (8)	60/65 (92)	<.001
Regurgitations	46 (68)	2/46 (4)	44/46 (96)	<.001
Difficulty swallowing	10 (15)	0/10 (0)	10/10 (100)	0.001

**Fig. 3:**
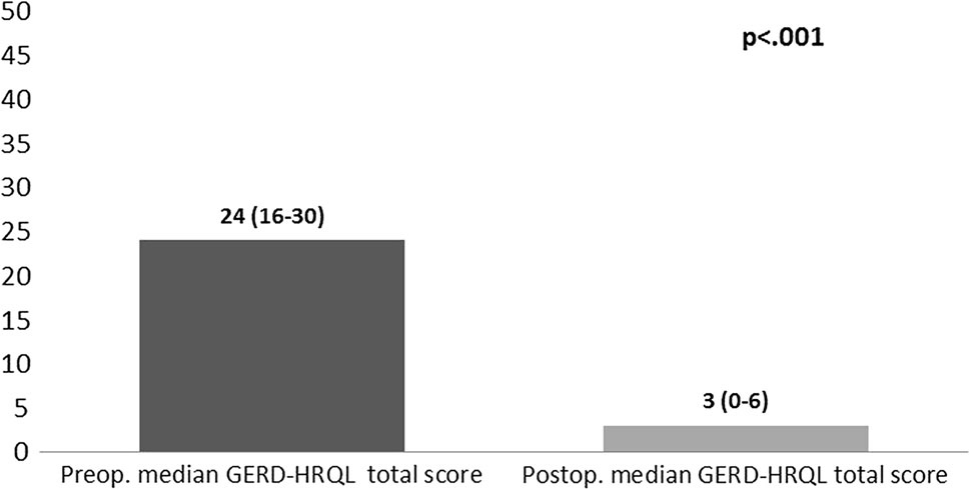
Comparison of pre- and postoperative median GERD-HRQL total scores (the maximum total score reachable is 50, with a lower score indicating a better QOL)

A standardized telephone interview was completed by 91% of patients (*n* = 62). The postoperative GERD-HRQL total score was significantly reduced after sphincter augmentation indicating significantly increased GERD-related Quality of Life [3 (IQR 0–6) vs. 24 (IQR 16–30); *p* < 0.001] (Fig. 3). Furthermore, the median alimentary satisfaction (AS) was excellent with 8/10.

Satisfaction with heartburn relief was achieved in 95% of patients. Ninety-five percent rated magnetic sphincter augmentation more effective than PPI use in terms of heartburn relief. PPI dependency was eliminated in 87% of patients. The overall postoperative outcome was specified as excellent/good by 89% of patients. Postoperative outcomes and Quality of Life results are shown in Table 3.

**Table 3:** Postoperative outcomes and Quality of Life results

	Total *n* = 62 (100%)
Median GERD-HRQL total score*	3 (IQR, 0–6)
Median alimentary satisfaction (AS)	8/10
Satisfaction with heartburn relief	59 (95)
Heartburn relief by MSA better than with PPIs^†^	59 (95)
Postop. outcome rated excellent/good	55 (89)
Postoperative PPI use^†^	8 (13)
Postoperative BMI^‡^	26 (23–29)

*GERD-HRQL total score ranges from 0 to 50; ^†^PPI(s) = proton pump inhibitors; ^‡^*BMI* body mass index

After a median follow-up time of 27.3 months (IQR 9–46), esophageal functioning tests (EFTs) were repeated in 34 patients (50%) (Table 4). Both, the median total percentage time pH < 4 [4.7 (IQR 2.6–10.3) vs. 0.9 (0.2–4.2), *p* = 0.003] and the median number of reflux episodes [67 (IQR 52–86) vs. 33 (20–42), *p* < .001] were significantly reduced after surgery. A normal DeMeester score was detected in 26 patients (76%), while it was abnormal (median 24.3 IQR 19.5–58) in 8 patients (24%). The total percentage time pH < 4 was normal in 27 patients (79%) and abnormal (median 7.1, IQR 6.45–13.1) in 7 patients (21%).

**Table 4:** Results of postoperative esophageal functioning testing (EFT)

	Total *n* = 34 (100%)
Time of surgery to EFT (months)	27.3 (9–46)
Negative pH results	27 (79)
Median total # reflux episodes (normal < 73)	33 (20–42)
Median total percent time pH < 4 (normal < 4.2%)	0.9 (0.2–4.2)
High-resolution manometry	
Normal esophageal motility	31 (91)
IEM*	3 (9)
Median LES resting pressure (normal 10–45 mmHg)	23 (17–27)
Median IRP^†^ (normal < 20 mmHg)	14 (12–19)

*IEM ineffective esophageal motility; ^**†**^*IRP* integrated relaxation pressure of LES

Ineffective esophageal motility (IEM) was seen on high-resolution motility in 3 cases (9%), and regular esophageal motility was seen in the remaining 31. The median LES resting pressure and IRP were within normal limits; however, 6 cases (18%) were classified as EGJ outflow obstructions (median IRP 27, range 23–36).

Twelve of the 15 patients (80%) with normal preoperative pH findings but positive symptom correlation were satisfied with their postoperative heartburn relief. The outcome was rated “excellent/good” by 67% (*n* = 10) of patients. Postoperative objective testing was performed in 10 of these patients (75%). A normal total percentage time pH < 4 was detected in 7 cases (70%), while it was abnormal in 3 patients (30%).

## Discussion

Magnetic sphincter augmentation (MSA) is a novel concept in the treatment of gastroesophageal reflux disease utilizing a ring of magnetic beads that augments the lower esophageal sphincter (LES) to minimize pathologic reflux. In the current study, we evaluated the outcomes and Quality of Life of GERD patients following magnetic sphincter augmentation at a specialized reflux center over a five-year period. We found sphincter augmentation to result in significant symptom relief, high postoperative satisfaction, significantly improved GERD-related Quality of Life and excellent alimentary satisfaction with low perioperative morbidity. Furthermore, a normalization of pH results was detected in 79% of cases following MSA.

In this series, sphincter augmentation led to significant relief of all major GERD symptoms. Heartburn was eliminated in 92% of patients. This is consistent with the previously reported rates of heartburn relief of 93.9% after LINX and 96.5% post-Nissen [23]. Satisfaction with heartburn relief was achieved in 95% of our patients, and 95% rated magnetic sphincter augmentation more effective than PPI use in terms of heartburn relief. Regurgitations were successfully eliminated in 96% of cases which matches findings of other groups where regurgitations were relieved in 93.9% after fundoplication and 100% post-MSA [23].

Preoperative dysphagia resolved in 100% of cases. None of our patients suffered from persistent dysphagia at the last follow-up. However, 16% of patients reported occasional difficulty swallowing with solids only while another 21% reported rare difficulties swallowing with solids only. Rates of mild dysphagia after MSA have been described to vary between 52 and 45%, while persistent dysphagia usually does not occur at the last follow-up [2, 7, 21, 24]. A total of 89% of patients stated they were satisfied with the overall postoperative outcome. Other studies reported no significant differences in overall satisfaction rates after MSA (77%) and fundoplication (78%) [25]. The dependence on PPIs was eliminated in 87% of our patients. According to literature, PPI use reportedly ranges between 8–20% and 8–14% after MSA and post-Nissen, respectively [1, 3, 6, 7, 13, 25, 26].

The postoperative GERD-HRQL total score was significantly reduced after surgery (3 (IQR 0–6) vs. 24 (IQR 16–30); *p* < 0.001) indicating a significantly improved GERD-related Quality of Life after MSA. This matches the observations of Bonavina et al. who reported a reduction from 25.7 to 3.3 of the mean GERD-HRQL total score four years after MSA (*p* < 0.001).

Furthermore, the current study showed an excellent alimentary satisfaction of 8/10, indicating that patients are comfortably eating after LINX implantation.

Objective postoperative evaluation by impedance-pH-metry was performed to assess reflux control provided by sphincter augmentation. Comparing pre- and postoperative results, both the median total percentage time pH < 4 [4.7 (IQR 2.6–10.3) vs. 0.9 (0.2–4.2), *p* = 0.003] and the median number of reflux episodes [67 (IQR2 52–86) vs. 33 (20–42), *p* < .001] were significantly reduced after surgery. Normal pH results were revealed in 79% of our patients. Comparable findings were reported by Bonavina et al., who described a negative pH rate of 75% five years after magnetic sphincter augmentation [13]. Comparing outcomes after MSA and fundoplication, previous studies found that laparoscopic fundoplication results in greater reduction of baseline percentage time pH < 4, lower acid exposure of the distal esophagus and higher rates of PPI cessation [23, 25]. This might be explained by the “two-sphincter theory” and the variable percentage of crural closure performed between groups [24, 27–29].

In this series, postoperative pH normalization could not be achieved in 7 patients (21%). None of them had a hiatal hernia larger than 4 cm; while 5 patients were treated by sphincter augmentation the other 2 received additional hiatal repair. Reasons for failure might have been the underlying obesity in three patients (43%; BMI range 28.4–32.5) and the ineffective esophageal motility (IEM) at time of surgery in one of them (14%). However, all 7 patients experienced great symptom relief and rated their postoperative outcomes as excellent/good.

Sphincter augmentation led to sufficient heartburn relief in 80% of patients (*n* = 12) with normal pH findings but positive symptom correlation for heartburn prior to surgery. Postoperative pH testing revealed normal results in 70% of cases. The remaining 30% of patients stated to be satisfied with their symptom relief despite abnormal pH findings. It is noteworthy that all 3 patients (20%) with unsatisfactory heartburn relief showed normal postoperative pH results.

This study included 3 cases (4%) of ineffective esophageal motility (IEM). Postoperative reflux control, heartburn relief and satisfaction with outcomes were comparable to those in patients with normal esophageal motility. None of the 3 patients developed postoperative dysphagia. To date, the surgical gold standard in the treatment of GERD patients with esophageal motility abnormalities is the laparoscopic Toupet fundoplication [30, 31]. To our knowledge, no study has been investigating the outcomes of magnetic sphincter augmentation in IEM patients. Our findings suggest that MSA could be a viable alternative to Toupet fundoplication in these patients. However, this hypothesis has yet to be proven.

No perioperative complications occurred, and there were no cases with device migration or erosion. Persistent postoperative dysphagia occurred in 2 patients (3%) who were successfully treated by endoscopic dilation. The LINX device was explanted in another 2 patients due to retrosternal pain and discomfort. Explantation rates reportedly range from 2.2 to 7% [7, 24]. While the pivotal trial reported that 19 of 100 MSA patients (19%) underwent dilatation for dysphagia; Bonavina et al. later described a dilation rate of only 2%. This might be explained by the fact that initially patients received a liquid diet after surgery, allowing for scar tissue to form. Nowadays, a solid diet is started on postoperative day 1 [13, 24].

The right preoperative patient selection is crucial and should be based on endoscopic evaluation followed by an assessment of reflux severity and esophageal motility. Acknowledging the learning curve, it is important that MSA is performed by experienced surgeons to achieve comparable results to those after fundoplication. Despite the low rate of side effects post-LINX, further prospective multicenter studies are necessary to investigate possible risk factors for the development of postoperative dysphagia and dissatisfying reflux control.

We are aware of the limitations of a retrospective study. Objective testing was available in only 50% of patients and was not performed at a standardized follow-up time. Further prospective studies with larger sample sizes and standardized pH testing at the one year follow-up would be of value.

## Conclusion

Magnetic sphincter augmentation is leading to significant symptom relief, increased GERD-specific Quality of Life and excellent alimentary satisfaction with low perioperative morbidity. This study adds important information to the growing evidence that magnetic sphincter augmentation should be considered an excellent alternative to fundoplication in the treatment of chronic reflux disease when performed by experienced surgeons.
